# Performance comparison of three deep learning models for impacted mesiodens detection on periapical radiographs

**DOI:** 10.1038/s41598-022-19753-w

**Published:** 2022-09-13

**Authors:** Kug Jin Jeon, Eun-Gyu Ha, Hanseung Choi, Chena Lee, Sang-Sun Han

**Affiliations:** Department of Oral and Maxillofacial Radiology, Yonsei University College of Dentistry, 50-1 Yonsei-ro, Seodaemun-gu, Seoul, 03722 Korea

**Keywords:** Dental diseases, Dentistry, Periapical radiographs, Mathematics and computing

## Abstract

This study aimed to develop deep learning models that automatically detect impacted mesiodens on periapical radiographs of primary and mixed dentition using the YOLOv3, RetinaNet, and EfficientDet-D3 algorithms and to compare their performance. Periapical radiographs of 600 pediatric patients (age range, 3–13 years) with mesiodens were used as a training and validation dataset. Deep learning models based on the YOLOv3, RetinaNet, and EfficientDet-D3 algorithms for detecting mesiodens were developed, and each model was trained 300 times using training (540 images) and validation datasets (60 images). The performance of each model was evaluated based on accuracy, sensitivity, and specificity using 120 test images (60 periapical radiographs with mesiodens and 60 periapical radiographs without mesiodens). The accuracy of the YOLOv3, RetinaNet, and EfficientDet-D3 models was 97.5%, 98.3%, and 99.2%, respectively. The sensitivity was 100% for both the YOLOv3 and RetinaNet models and 98.3% for the EfficientDet-D3 model. The specificity was 100%, 96.7%, and 95.0% for the EfficientDet-D3, RetinaNet, and YOLOv3 models, respectively. The proposed models using three deep learning algorithms to detect mesiodens on periapical radiographs showed good performance. The EfficientDet-D3 model showed the highest accuracy for detecting mesiodens on periapical radiographs.

## Introduction

Mesiodens, which refers to a supernumerary tooth that occurs in the maxillary central incisor region, is the most common type of supernumerary tooth^1^. Impacted mesiodens can cause delayed eruption, displacement, rotation, and root resorption of adjacent teeth, as well as crowding, diastema, and cysts in children^2^. An accurate diagnosis of mesiodens reduces complications and the extent of surgical or orthodontic treatment by enabling the most appropriate and minimal treatment^3^. Periapical radiographs, occlusal radiographs, and panoramic radiographs have been used to analyze the shape and location of mesiodens and to evaluate the presence of complications^4^.

Panoramic radiography was widely used for screening and diagnosis in dental clinic. But in pediatric patients, the risk of radiation-induced damage is two to three times higher than in adults^5^. According to the American Dental Association (ADA), selected periapical radiography is a guideline (https://www.fda.gov/media/84818/download)^6^ and dentists also prefer periapical radiography to panoramic radiography to evaluate new pediatric patients^7^. Periapical radiography is frequently used in children for the diagnosis of delayed eruption, trauma, dental caries, and orthodontics in the maxillary anterior region. On periapical radiographs, it is sometimes difficult to detect impacted mesiodens due to the overlap of the anterior nasal spine and permanent teeth or inadequate central ray angles (e.g., horizontal or vertical angles), and inexperienced clinicians in particular may not be able to detect mesiodens. Developing an automated mesiodens detection model that is accurate and does not require any manual processes would be helpful for dental clinicians to diagnose mesiodens at an early stage.

In dentistry, artificial intelligence (AI) models have been introduced that use panoramic radiographs^8,9^ or cone-beam computed tomography (CBCT)^10,11^ to make automatic diagnoses. A few studies have investigated tooth identification^12,13^ and dental caries^14^ using periapical radiographs, but no studies have focused on detecting mesiodens.

Active research using AI-based models is underway in numerous scientific fields; in particular, research on AI detection models using the YOLO, RetinaNet, R-CNN, and SSD algorithms, among others, is advancing. YOLO and RetinaNet, which are deep learning algorithms with convolutional neural networks, have shown good results in detecting lesions or teeth^15,16^. The EfficientDet algorithm has been used to detect lesions on medical images^17,18^, but has rarely been used in dentistry. In this study, we utilized the EfficientDet algorithm, which has rarely been used in dentistry, as well as the YOLOv3 and RetinaNet algorithms, which have widely been adopted in dental research, for automatic mesiodens detection on periapical radiographs.

This study aimed to develop deep learning models that automatically detect impacted mesiodens on periapical radiographs of primary and mixed dentition using three deep learning algorithms and to compare the performance of the developed models.

## Materials and method

### Subjects

This study was approved by the Institutional Review Board (IRB) of Yonsei University Dental Hospital (No. 2-2021-0102) and was performed in accordance with ethical regulations and guidelines. The requirement for informed consent was waived by the IRB since this was a retrospective study and all data were used after anonymization.

Periapical radiographs of 720 patients (age range, 3–13 years) who visited Yonsei University Dental Hospital were collected between October 2018 to September 2021 in bitmap format. These 720 patients with primary and mixed dentition consisted of 660 patients with impacted mesiodens and 60 patients without mesiodens. All patients with impacted mesiodens underwent both periapical radiography and CBCT examinations, and the presence of mesiodens was confirmed through CBCT images. The periapical images were acquired from a Kodak 2200 Intraoral X-ray System (Eastman Kodak Co., NY, USA). The training and validation datasets consisted of 540 and 60 periapical images with mesiodens, and the test dataset consisted of 60 periapical images with mesiodens and 60 periapical images without mesiodens (Table 1).

**Table 1:** Number of periapical radiographs in the training, validation, and test datasets.

Group	Training	Validation	Test	Total
With mesiodens	540	60	60	660
Without mesiodens	–	–	60	60

### Development and evaluation of the model

**Figure 1:**
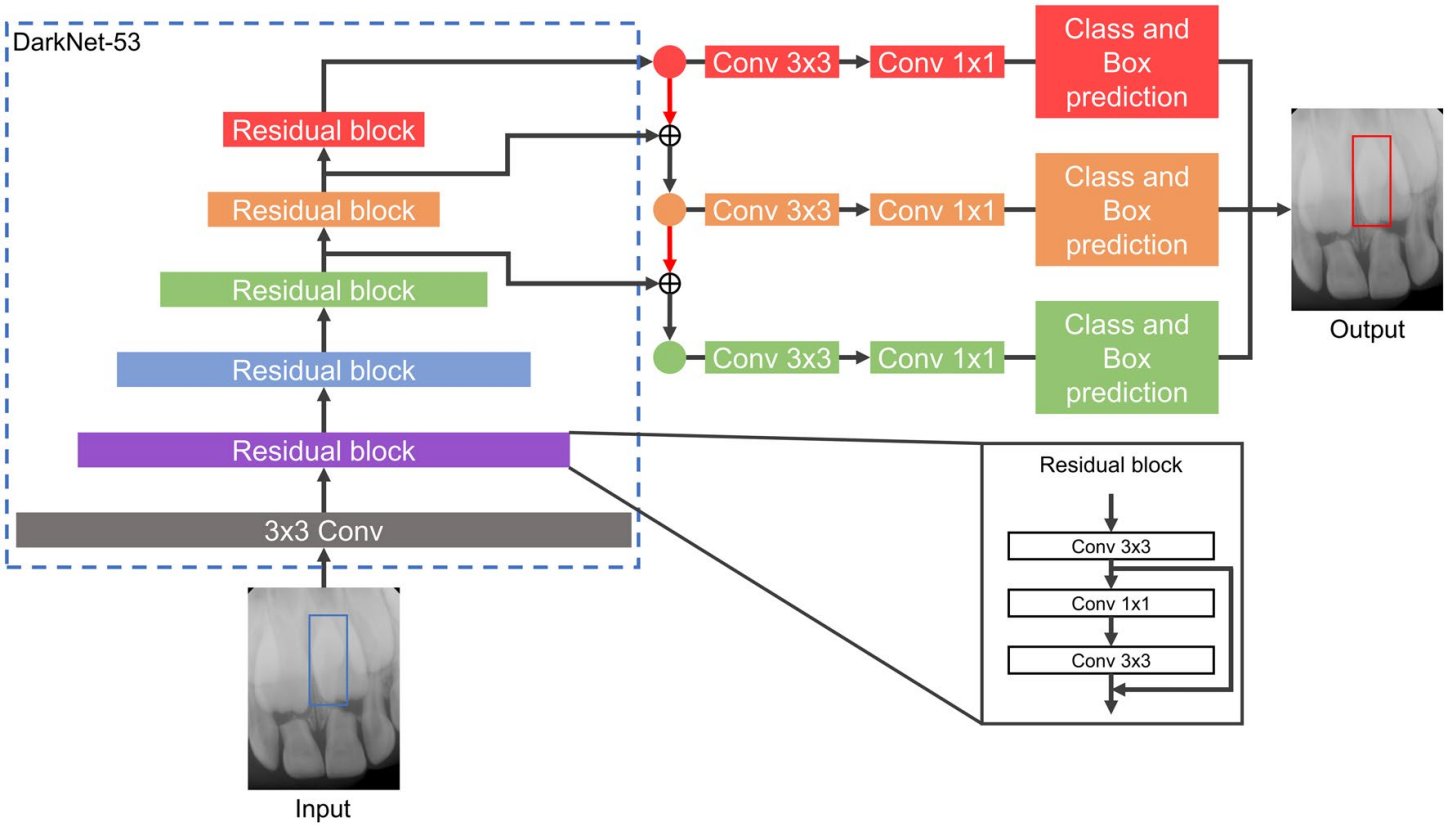
The structure of the YOLOv3 algorithm. *Conv* convolutional layer.

**Figure 2:**
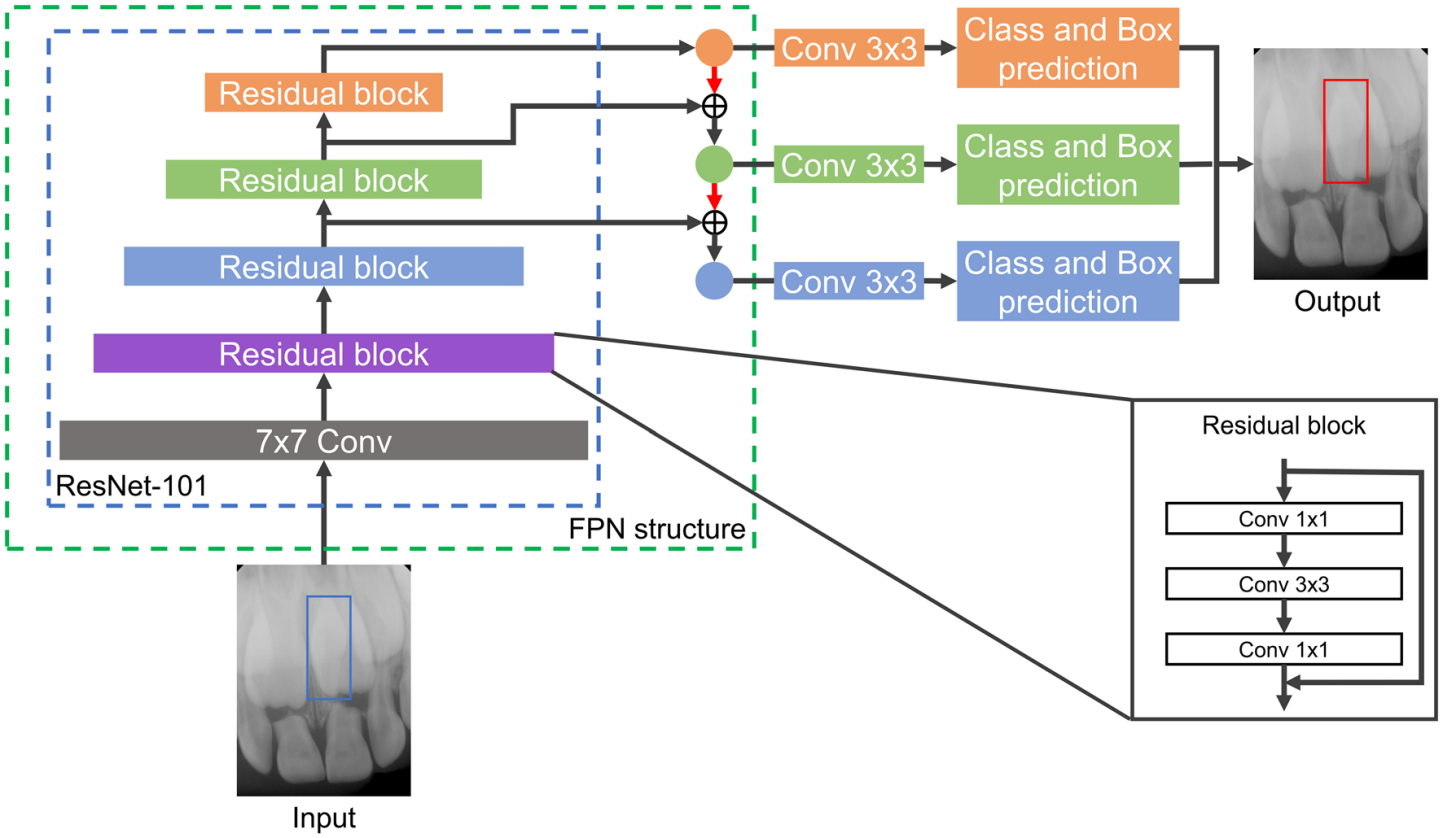
The structure of the RetinaNet algorithm. *FPN* feature pyramid network, *Conv* convolutional layer.

**Figure 3:**
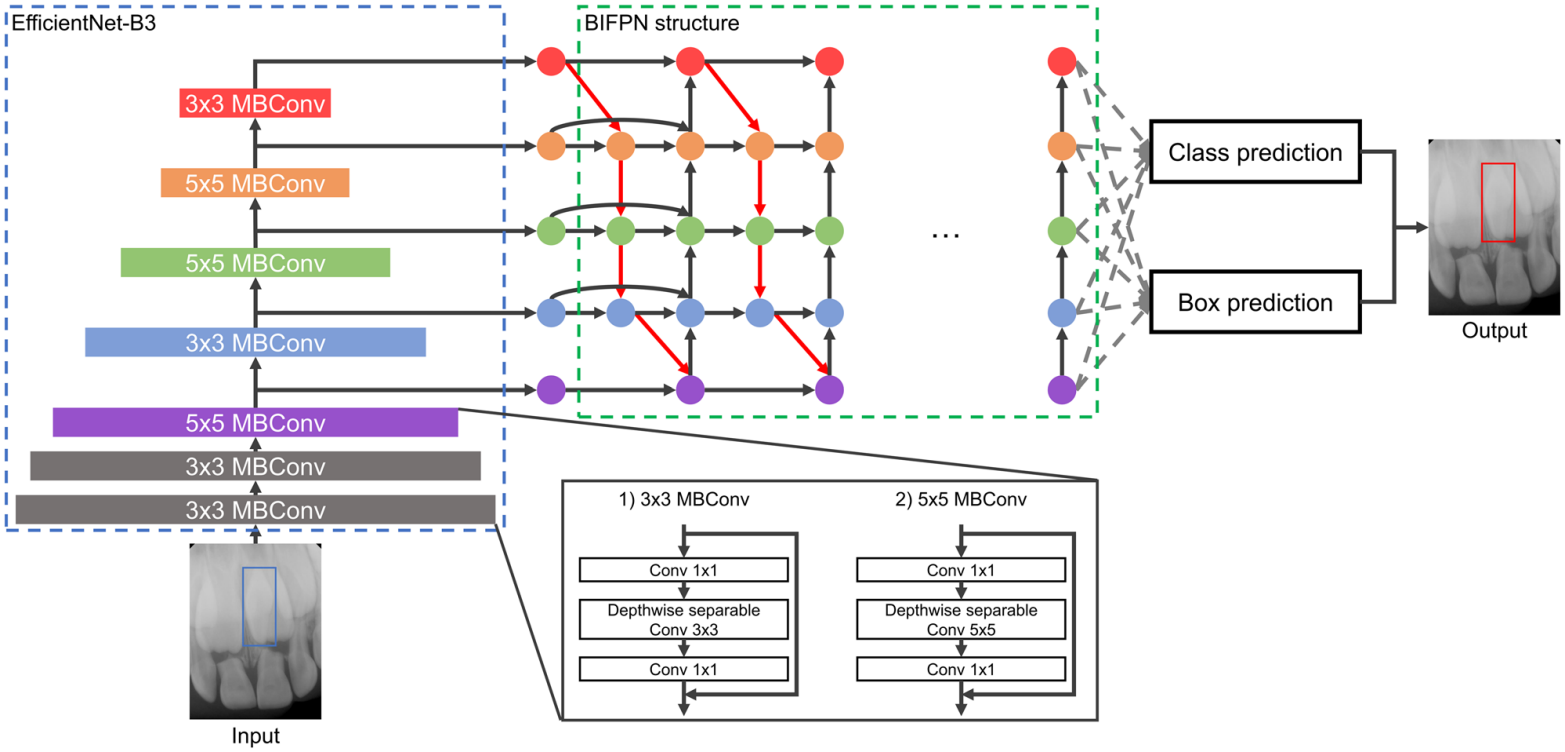
The structure of the EfficientDet-D3 algorithm. *BIFPN* bi-feature pyramid network, *MBConv* mobile inverted bottleneck convolution block.

The mesiodens detection models were developed with three deep learning algorithms: YOLOv3, RetinaNet, and EfficientDet-D3. The YOLOv3 algorithm, which was proposed by Redmon et al.^19^, uses darknet-53 as a backbone and binary cross-entropy loss as a loss function. The performance of YOLOv3 has the advantage of a fast inference time, as previous studies have verified in dental and medical images^20–22^. The RetinaNet algorithm, which was introduced by Lin et al.^23^, is characterized by detecting objects at various resolutions using ResNet and a feature pyramid network (FPN) structure as a backbone. This algorithm was the first to suggest using focal loss, which is a loss function focused on training on difficult cases by multiplying the cross-entropy loss by weight. The RetinaNet algorithm takes a longer inference time than the YOLOv3 algorithm, but has higher accuracy^19^. EfficientDet, which was developed by Tan et al.^24^, contains eight model structures (EfficientDet-D0–EfficientDet-D7). Due to the limitation of computing resources, EfficientDet-D3 with EfficientNet-B3 as a backbone was used in this study. The loss function of EfficientDet-D3 is focal loss, as in RetinaNet, but EfficientDet-D3 utilizes a bi-FPN structure that can detect objects at more diversely combined scales than RetinaNet’s FPN structure. Although EfficientDet-D3 has shown improved performance and efficiency compared to other object detection algorithms^24^, it has not yet been applied in the dental field. The structures of the three algorithms are shown in Figs. 1, 2, and 3, respectively.

**Figure 4:**
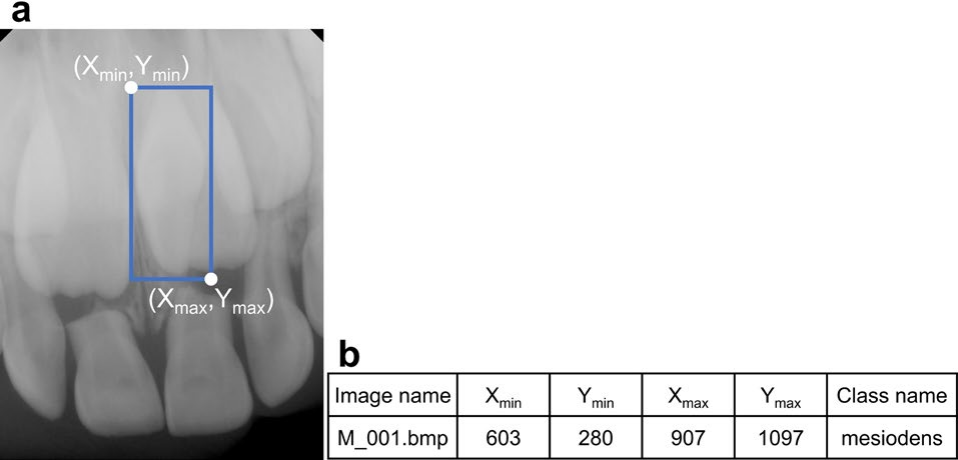
Example of an annotated periapical radiograph with a blue rectangular box (**a**) and extracted annotation information (**b**). The annotation information was composed of the upper left (X_min_, Y_min_), the lower right (X_max_, Y_max_), and the class name (“mesiodens”).

These models needed information on the training and validation dataset (i.e., the location and class name of the ground truth). An oral radiologist with 20 years of experience used the graphical image annotation tool LabelImg (version 1.8.4, available at https://github.com/tzutalin/labelImg) to manually annotate a rectangular region of interest (ROI) containing just the mesiodens as a gold standard for the training and validation datasets. Based on this annotation, the coordinates of the upper left (X1, Y1) and lower right (X2, Y2) corners of the ROI surrounding the mesiodens were extracted for the training and validation datasets (Fig. 4). The annotation information, along with the input periapical images, was used in the model training process.

All models were trained 300 times with our dataset using pre-trained weights from the coco dataset as initial weights. In the test process, when the trained models detected mesiodens on the input periapical radiograph, they provided an image marked with a red box in the detected area, and if they did not detect mesiodens, they provided the input periapical image without a box. An accurate prediction of mesiodens was defined as an intersection over union (IOU) value of the detected mesiodens region of 0.5 or higher^25^. The performance of the three models was evaluated and compared based on accuracy, sensitivity, specificity with the test dataset. Also, the precision-recall curve, which is a plot of recall (x-axis) and the precision (y-axis), was drawn based on the test results. All experiments were implemented in Windows 10 with Keras and TensorFlow deep learning frameworks using an NVIDIA TITAN RTX graphics card.

## Results

Table 2 shows the accuracy, sensitivity, and specificity of the three models using the test dataset. The YOLOv3 model showed an accuracy of 97.5%, a sensitivity of 100%, and a specificity of 95.0%. The RetinaNet model achieved an accuracy of 98.3%, a sensitivity of 100%, and a specificity of 96.7%. The EfficientDet-D3 model achieved the highest accuracy of 99.2%, a sensitivity of 98.3%, and a specificity of 100%.

**Table 2:** Accuracy, sensitivity, and specificity of the three models (%).

Evaluation index	Detection model		
	YOLOv3	RetinaNet	EfficientDet-D3
Accuracy	97.5	98.3	99.2
Sensitivity	100	100	98.3
Specificity	95.0	96.7	100

**Figure 5:**
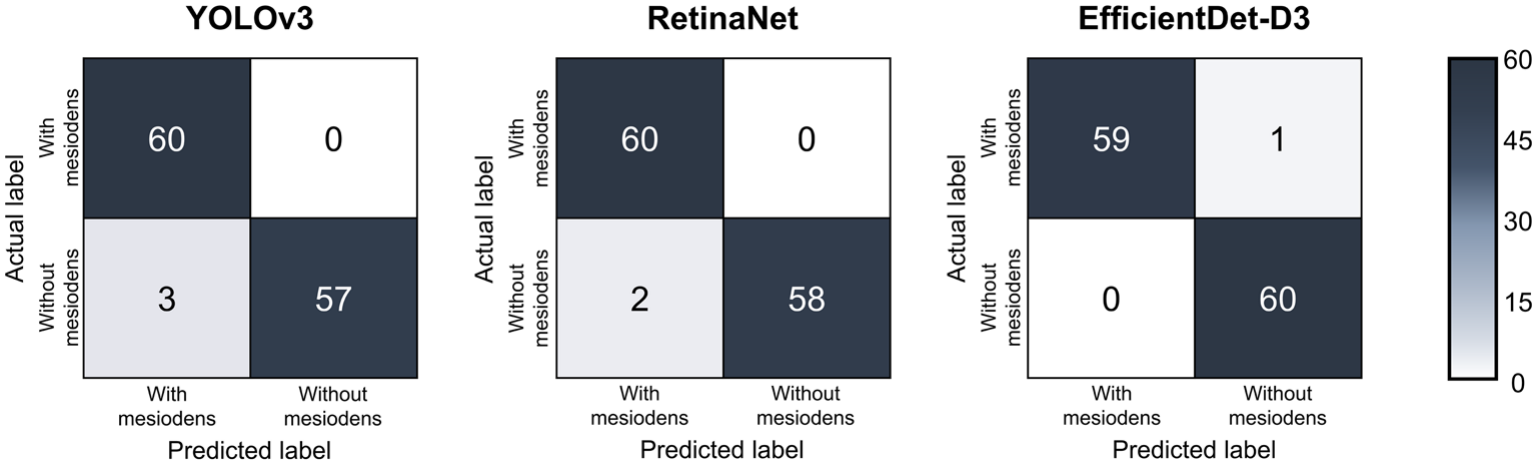
Confusion matrices of the test dataset for the three models.

**Figure 6:**
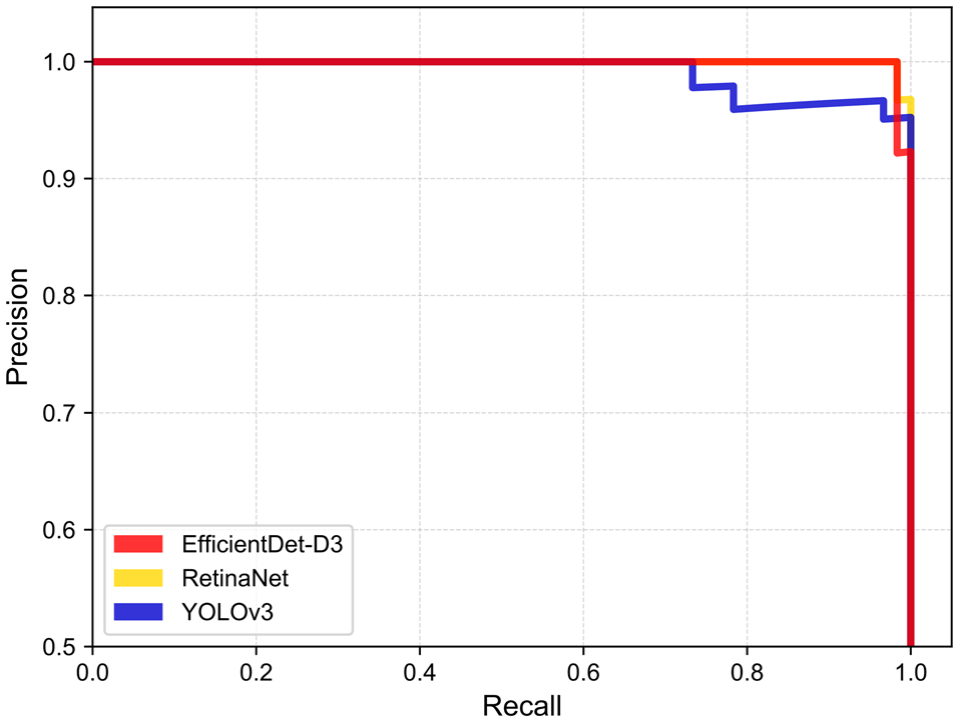
Precision-recall curves of the test dataset for the three models.

Confusion matrices and precision-recall curves of the three models using the test dataset are shown in Figs. 5 and 6.

**Figure 7:**
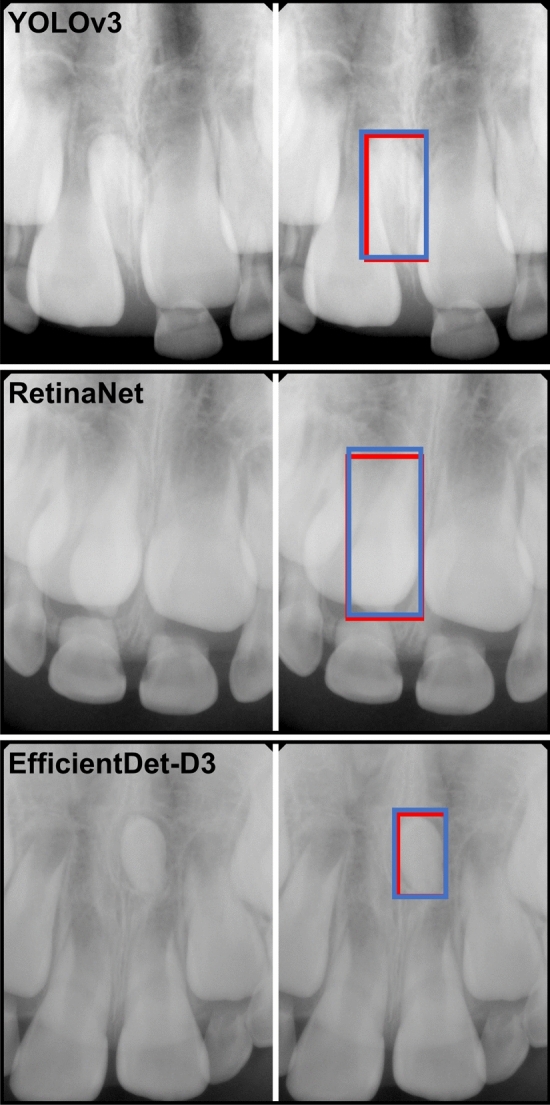
Examples of correctly detected mesiodens in the YOLOv3, RetinaNet, and EfficientDet-D3 models. The left side of each case is the original periapical radiograph, and the right side is the result of each model. The manually annotated label by the radiologist is shown as the blue box and the automatically detected mesiodens is shown as the red box.

**Figure 8:**
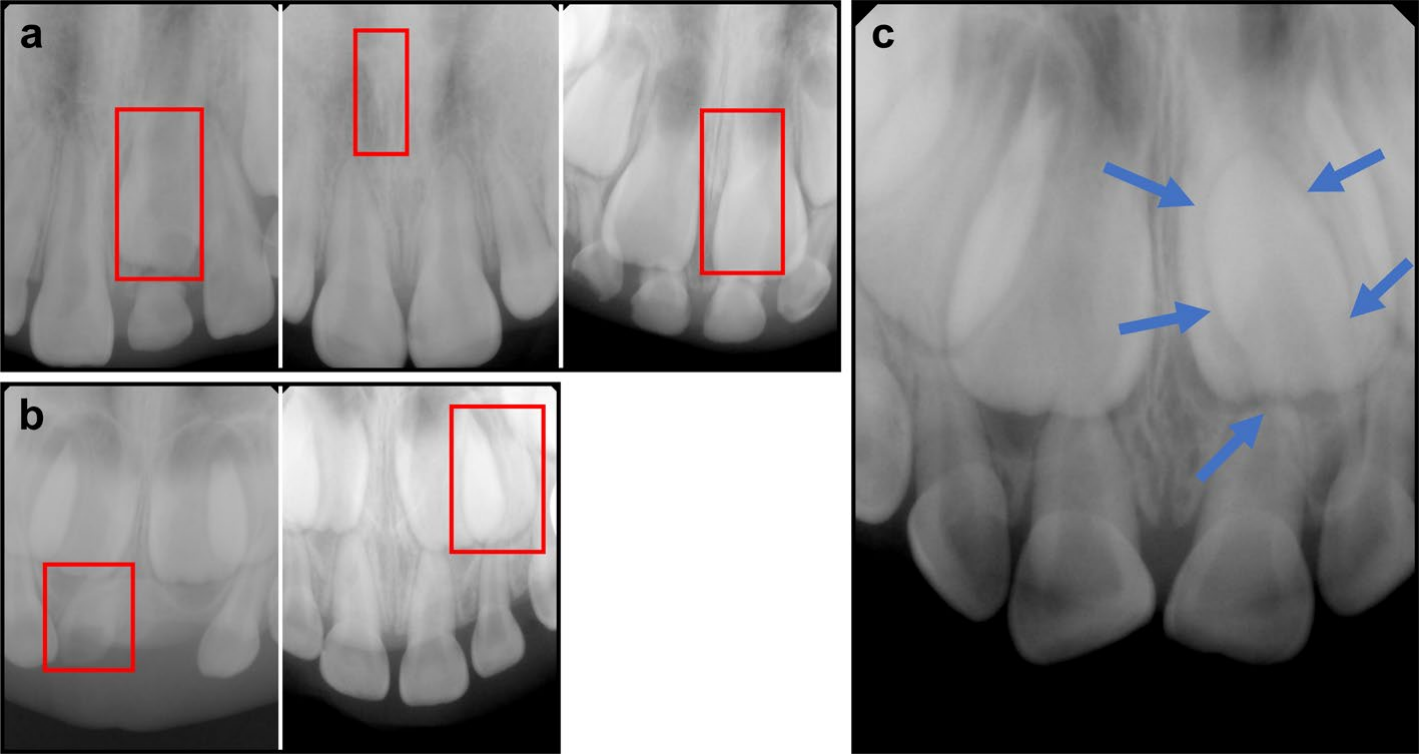
False-positive cases and a false-negative case. False-positive cases misdiagnosed as mesiodens in the YOLOv3 model (**a**) and the RetinaNet model (**b**), and a false-negative case in the EfficientDet-D3 model (**c**). Red boxes indicate the regions that were incorrectly detected as mesiodens, while blue arrows indicate undetected mesiodens.

Figure 7 presents the cases in which mesiodens was correctly detected by the three models. Figure 8 shows the false-positive and false-negative cases. The incorrect detection cases were confused with the anterior nasal spine, deciduous teeth, or permanent teeth.

## Discussion

Studies using AI models on various dental radiographic images have been actively conducted to confirm their potential as diagnostic aids. Deep learning algorithms are most commonly applied to panoramic radiographs^8,9^, CBCT^10,11^, and computed tomography (CT)^26,27^, and there have been relatively few studies applying deep learning models to periapical radiographs. Those studies have focused on tooth detection and numbering, tooth segmentation, and dental caries classification^12–14^. In the tooth detection and numbering model, precision and recall values of over 90% were obtained^13^, and in the tooth segmentation model, the dice similarity coefficient value was 0.95^12^. The accuracy of the model for classifying the presence of dental caries was 86.3%^14^. Since the field of view of periapical radiographs is small and image distortion, such as elongation, shortening, and overlapping depending on the angle of the central ray, can occur, studies on lesion diagnosis have been limited, and no study has yet dealt with supernumerary teeth. AI-based mesiodens diagnosis models using panoramic radiographs have been developed in several studies^15,28^. Our previous study^15^ based on panoramic radiographs reported that an automatic mesiodens detection model using the YOLOv3 algorithm showed an accuracy of 96.2%. However, for children with primary and mixed dentition, periapical radiographs and bitewing radiographs are usually recommended, rather than panoramic radiographs; therefore, it might be more valuable to use periapical images for mesiodens detection.

This study is the first attempt to develop deep learning models to detect mesiodens on periapical radiographs, and it compared the accuracy of models using the YOLOv3, RetinaNet, and EfficientDet-D3 algorithms. EfficientDet-D3, which was used for dental radiographs for the first time, showed the best accuracy among the three AI models. The YOLOv3 model is the most representative deep learning model used for object detection, and it has been widely applied with good results in medical and dental images^15,16,29^. The RetinaNet has a longer processing time but higher accuracy than YOLOv3, and it has also proved its usefulness in medical and dental imaging^30,31^. As a new family of object detection algorithms, the EfficientDet model has been applied in various fields such as manufacturing and agriculture^32–34^, where it has achieved state-of-the-art performance. It is a high-efficiency algorithm that has a small size and fast inference time and shows high accuracy with a relatively low amount of computation compared to other object detection algorithms^24^. In the medical field, the EfficientDet algorithm was used for the analysis of blood smears^35^ and the detection of diabetic foot ulcers^18^, and it showed excellent performance (97.9% accuracy) in detecting glaucoma in fundus images^17^. However, it has not yet been applied in the dental field. We confirmed that the YOLOv3, RetinaNets and EfficientDet-D3 models showed high accuracy (97% or higher) in detecting mesiodens. The sensitivity was higher in the YOLOv3 and RetinaNet models, and the specificity was higher in the EfficientDet-D3 model. The EfficientDet-D3 model had the highest overall accuracy.

The present study has limitations in that it only dealt with periapical radiographs taken using one device at one institution and did not include multiple mesiodens cases. In further research, collecting periapical radiographs from multiple institutions with several different X-ray machines and including patients with two or more mesiodens would improve the performance of the model, at which point it would have potential for clinical use in the dental field.

## Conclusion

Automated mesiodens detection models based on periapical radiographs were developed using three deep learning algorithms (YOLOv3, RetinaNet and Efficientdet-D3) in this study. All models showed an accuracy of 97% or higher, and the EfficientDet-D3 model showed the highest accuracy, confirming that it is a useful algorithm for dental radiographs.

## Data Availability

The data generated and analyzed during the current study are not publicly available due to privacy laws and policies in Korea, but are available from the corresponding author on reasonable request.
